# Totally thoracoscopic surgery for treating left atrial myxoma

**DOI:** 10.1097/MD.0000000000027819

**Published:** 2021-11-12

**Authors:** Feng Zhao, Tongyun Chen, Yipeng Tang, Qingliang Chen, Nan Jiang, Zhigang Guo

**Affiliations:** Department of Cardiac Surgery, Tianjin Chest Hospital, Tianjin, China.

**Keywords:** cardiac tumor, left atrial myxoma removal surgery, minimally invasive cardiac surgery, totally thoracoscopic surgery

## Abstract

We aimed to summarize the experience of totally thoracoscopic surgery for left atrial myxoma, together with analyzing the safety and feasibility. We retrospectively analyzed the clinical data of 15 patients with left atrial myxoma admitted to our hospital from October 2016 to October 2018. The auxiliary hole was located at the midline of the 5th intercostal space of the right chest. The endoscope hole was located at the front position of the fourth intercostal space. Specimens were sent to the pathology department for pathological examination. All the procedures were completed successfully. Extracorporeal circulation time was 46.5 ± 18.6 minute, cross-clamping time was 20.6 ± 6.7 minute, thoracic drainage fluid was 89+60.2 ml, ventilator assist time was 4.3 ± 2.6 hour, intensive care unit stay time was 14.5 ± 4.2 hour, the average postoperative hospital stay was 5.2 ± 1.2 day. There was no death, or red blood cell transfusion during and after surgery. No postoperative complications were reported by the patients. No recurrence of myxoma, residual shunt in the atrial septum and valvular lesions were found after 3months of postoperative cardiac ultrasound examination. Total thoracoscopic surgery for left atrial myxoma was less invasive with satisfactory cosmetic appearance with feasibility and safety. Besides, it caused no serious complications.

## Abstract 2

Supplemental Digital Content is available in the text

## 1 Introduction

Cardiac myxoma, a common type of cardiac benign tumor, usually occur in the individuals aged between 30 and 50 years. It may take place in each cardiac ventricles, especially the left atrium followed by the right atrium. In rare conditions, some patients may present myxoma in the cardiac ventricle. Upon confirmation of the cardiac myxoma, surgery should be carried out to resect the tumor to restore the cardiac function. Conventional excision of myxoma in left atrium usually involves a median incision on the sternum. The sternum was dissected in the longitudinal pattern, which involves a large trauma and a length of incision of about 20 cm. In addition, the thoracic cage stability was hampered due to separation of the sternum, together with a longer duration post-surgery. Recently, microinvasive techniques have been commonly utilized in the treatment of atrial myxoma including mini-thoracotomy cardiac surgery, thorascopic surgery, and robot.^[1–3]^ Nevertheless, there are still some disputes on the feasibility and safety of the microinvasive techniques especially the resection of left atrium myxoma under the thoracoscope. In this study, we retrospectively analyzed the clinical data of the 15 cases with left atrial myxoma admitted to our hospital between October 2016 and October 2018. We aim to investigate the safety and feasibility of the left atrial myxoma under thorascope, in order to provide clinical guidance to the surgical options for the left atrial myxoma.

## 2 Materials and methods

### 2.1 Patients

**Figure 1:**
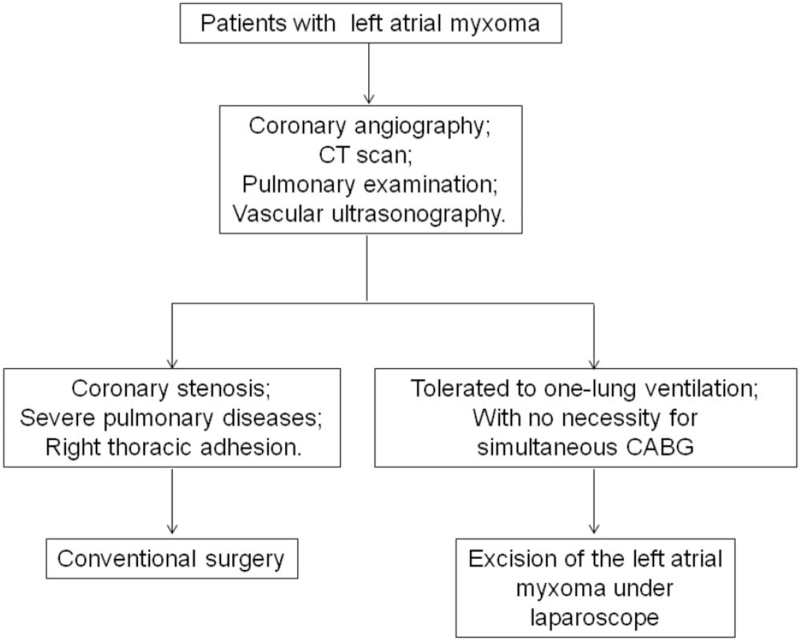
Study flowchart. CABG = coronary artery bypass grafting.

The patients with left atrial myxoma admitted to Tianjin Chest Hospital between October 2016 and October 2018 were included in this study. All the patients received ultrasonographic examination on the femoral artery and femoral veins, besides coronary angiography, CT scan and pulmonary examination based on the flowchart (Fig. 1). The inclusion criteria were as follows: those with preoperative echocardiogram indicated space-occupying lesions in left atrium; those with no severe coronary stenosis, severe lung diseases, or right thoracic adhesion, as well as those with no severe calcification or stenosis in the femoral arteries and veins. The pedicle of the tumor was localized in the interatrial septum. Those received simultaneous coronary artery bypass grafting, with severe lung diseases, right thoracic adhesion or no tolerance to the left-sided ventilation, or those with severe organ failure were excluded from this study. There were 15 patients (male: 6; female: 9) with left atrial myxoma admitted were included. The average age was 47.8 ± 10.5, whereas the body weight was 67.8 ± 20.6 kg. Eleven showed a cardiac function of grade II (NYHA), whereas the other 4 showed a grade of III. Six showed hypertension, and 2 showed diabetes mellitus. One patient showed cerebral infarction before surgery (Table 1). We obtained the written informed consent from each patient. The study protocols were approved by the Ethics Committee of Tianjin Chest Hospital.

**Table 1:** Patient characteristics.

Variables	Results
Case number	15
Male/female	6/9
Age, yrs	47.8 ± 10.5
Body weight, kg	67.8 ± 20.6
NYHA classification, I/II/III/IV	0/11/4/0
Hypertension	6
Diabetes mellitus	2
Cerebral infarction	1
Atrial fibrillation	0
Tumor diameter, cm	5.3 ± 4.6
Extracorporeal circulation duration, min	46.5 ± 18.6
Aortic blockage time, min	20.6 ± 6.7
Volume of chest drainage, ml	89 ± 60.2
ICU stay, h	14.5 ± 4.2
Postoperative hospital stay, d	5.2 ± 1.2

ICU = intensive care unit.

### 2.2 Surgery

**Figure 2:**
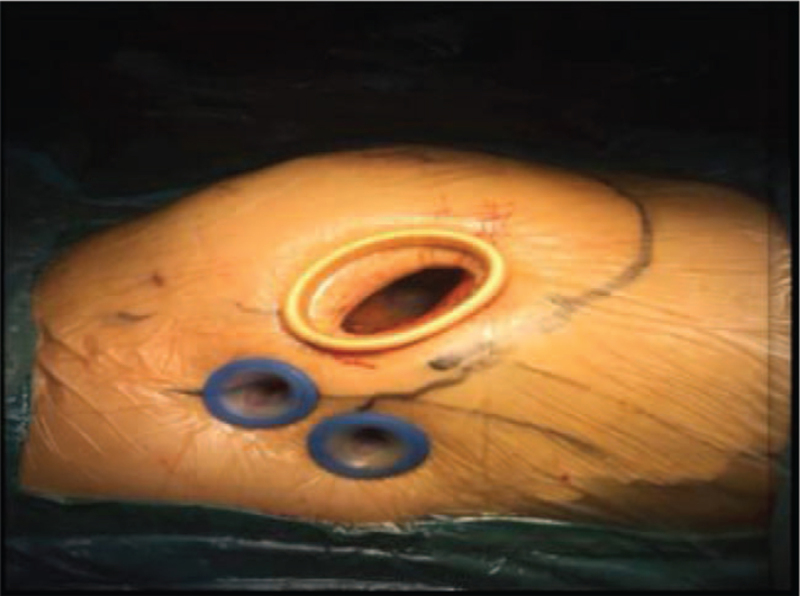
Position of the main operating hole, auxiliary hole, and endoscope hole.

The patient received trachea cannula, and was in a supine position after balanced anesthesia. Cannula was inserted into the right internal jugular vein for the drainage of the superior vena cava. Extracorporeal circulation was established through the femoral arterial cannulation and venous cannulation. An incision (3 cm) was made on the right thoracic wall which was localized in the 4^th^ midclavicular line. The satellite hole was localized at the right side of the 5^th^ midaxillary line. The surgery was performed according to the previous description with slight modifications. Briefly, the procedures were fully illustrated in the Supplementary Video, Supplemental Digital Content, http://links.lww.com/MD2/A647. The position of the main operating hole, auxiliary hole and endoscope hole were illustrated in Figure 2.

## 3 Results

All the patients accomplished the surgery successfully, and no one was transferred to the thoracotomy. The tumor diameter was about 5.3 ± 4.6 cm. The extracorporeal circulation duration was about 46.5 ± 18.6 minute. The duration for the aortic blockage was about 20.6 ± 6.7 minute. The volume of chest drainage after surgery was 89 ± 60.2 ml. The duration for the assistance of respirator after surgery was about 4.3 ± 2.6 hour. No red blood cell transfusion was required during and after surgery. The stay in the intensive care unit was about 14.5 ± 4.2 hour. The postoperative hospital stay was 5.2 ± 1.2 day (Table 1). There was no death in this group. After surgery, no hemostasis after thoracotomy, hematoma in the cannula site, poor healing, or abnormalities in the movement of lower limbs were noticed. One patient showed coronary artery embolization, and was recovered after embolism therapy via coronary artery. The patients were all followed up for at least 3 months after surgery. Cardiac ultrasonography indicated no recurrence of myxoma, no residual shunt in the interatrial septum and valvular heart disease.

## 4 Discussion

Cardiac myxoma is one of the most common type of primary benign tumors in clinical settings. Echocardiography is considered as a convenient and safety way for the diagnosis of left myxoma. It could provide helpful information to the profile, activity and adhesion of tumor pedicle, atrioventricular valve injury and ventricular size. The common symptoms of left atrial myxoma are associated with the palpitation and shortness of breath induced by blockage of atrioventricular blood. The myxoma with a larger mobility, such as sudden blockage of atrioventricular orifice, may induce faint and even sudden death to the patients. Myxoma patients may present develop cerebral embolism. Therefore, for the patients confirmed with left atrium myxoma, surgery should be carried out as soon as possible.^[4]^

In 1954, Chitwood et al^[5]^ accomplished the resection of left atrial myxoma. In 1998, Ko et al^[6]^ reported the excision of left atrial myxoma in 3 cases via right-sided micro-incision. With the development of the thoracoscope, Panos et al^[7]^ reported the left atrial myxoma resection, in which 10 patients accomplished the surgery. During the surgery, there were no conditions that may lead to termination of surgery, and the postoperative recovery was satisfactory. In China mainland, the first surgery for resection of cardiac myxoma under the thoracoscope.^[8]^ All these demonstrated that mini-invasive surgery contributed to the safety and feasibility of the left atrial myxoma.

The conventional surgery for treating left atrial myxoma is mainly relied on the median incision on the chest, which involves the opening of the sternum. The surgical wound was large. The postoperative pain and thoracic cage stability could affect the respiration, cough and sputum-exclusion. In addition, the patient showed nonunion after surgery, together with foreign body reaction. Meanwhile, the incision was long, and the cosmetic appearance may lead to a mental burden. Compared with the conventional surgery, left atrial myxoma under the thorascope showed small surgery-related wound, together with blood loss and postoperative attenuation in the surgery and rapid recovery. This was in line with the attenuation of surgery trauma, which contributed to the rapid recovery. On one hand, there is no need to open the sternum under the thoracoscope, and only an incision with a length of about 3 cm was made on the right thoracic wall. The surgery was performed along the ribs. The osseous structure was preserved to some extent, in order to reduce the risk of adverse events induced by nonunion and wire fixation. On the other hand, the thoracic stability was well preserved. The respiration function of the patients received thoracoscope was also preserved. Moreover, postoperative pain showed attenuation to some extent, which could reduce the irritability. This contributed to the attenuation of the symptoms such as cough, sputum-exclusion and respiratory function recovery. Moreover, patients could walk after removal of intrathoracic drain tube, and the movement time was longer than that of the conventional surgery. It contributed to the postoperative recovery, which can avoid phlebothrombosis in the lower limbs and reduce the hospital stay and cost.

For the surgery of left atrial myxoma under thoracoscope, we summarized the clinical experiences of the 15 cases: the patient with myxoma showed fragile texture and easy abscission. Special attention should be paid to the rupture and desquamation of the tumor mass in the surgery. For the management of tumor pedicle, intact removal of tumor pedicle and adhesion parts should be conducted. The depth and scale was at least 0.5 cm. Then the interatrial septum showed defect to some extent. On this basis, direct suturing and autologous pericardium were utilized to repair the interatrial septum. During the surgery, the ultrasonographic probe was inserted via the esophagus, which was used for the real-time monitoring of cardiac valve conditions.

Compared with the conventional methods, excision of the left atrial myxoma under thoracoscope involved longer extracorporeal circulation duration and block of aorta. However, the longer extracorporeal circulation (46.5 ± 18.6 minute) duration and block of aorta (20.6 ± 6.7 minute) did not bring in more adverse events and complications. In our study, patients underwent such procedures showed rapid recovery in the respiratory function as less trauma was induced to the pericardium, together with less bleeding and drainage requirement. However, for the patients with severe coronary stenosis before surgery, conventional incision on the middle chest was required to facilitate the coronary artery bypass grafting, especially for the exposure of the anterior descending branch and the convolution branch. Additionally, for the patients with severe femoral arterial stenosis, right thoracic adhesion or with poor tolerance of respiratory function to the one-lung ventilation, they were not suitable for the excision of the left atrial myxoma under thoracoscope. The Robotic technique involves mini-invasive procedures, however, the cost is really high.^[9,10]^ Compared with these studies, the total thoracoscopic surgery performed by our team is less than robotic surgery in terms of extracorporeal circulation time, aortic block time, intensive care unit stay time, and hospital stay. The thoracoscope technique shows not much difference compared with the conventional technique, and involves less cost compared with the robotic technique.

Indeed, there are some limitations in this study. The sample size was relatively small, and all the cases only received excision of the left atrial myxoma under thoracoscope, and no management was given to the bicuspid valve or the tricuspid valve, or radiofrequency ablation to the patients with atrial fibrillation. In future, studies of a large sample size are required to further illustrate the experiences on the excision of the left atrial myxoma under thoracoscope.

In summary, excision of the left atrial myxoma under thoracoscope contributed to a satisfactory cosmetic appearance with less severe complications. The postoperative recovery was satisfactory, and the treatment efficiency was good. Therefore, left atrial myxoma resection was feasible and safe.

## Author contributions

**Conceptualization:** Feng Zhao.

**Data curation:** Feng Zhao, Yipeng Tang.

**Formal analysis:** Yipeng Tang.

**Funding acquisition:** Feng Zhao.

**Investigation:** Qingliang Chen.

**Methodology:** Tongyun Chen, Qingliang Chen.

**Project administration:** Tongyun Chen.

**Resources:** Tongyun Chen.

**Software:** Tongyun Chen.

**Validation:** Qingliang Chen.

**Visualization:** Zhigang Guo.

**Writing – original draft:** Feng Zhao.

**Writing – review & editing:** Nan Jiang, Zhigang Guo.
